# Exploring non-participation in primary care physical activity interventions: PACE-UP trial interview findings

**DOI:** 10.1186/s13063-016-1299-z

**Published:** 2016-04-01

**Authors:** Rebecca Normansell, Rebecca Holmes, Christina Victor, Derek G Cook, Sally Kerry, Steve Iliffe, Michael Ussher, Julia Fox-Rushby, Peter Whincup, Tess Harris

**Affiliations:** Population Health Research Institute, St George’s, University of London, Cranmer Terrace, London, SW17 ORE UK; Gerontology and Health Services Research Unit, Brunel University, London, UB8 3PH UK; Pragmatic Clinical Trials Unit, Queen Mary University of London, London, E1 2AT UK; Research Department of Primary Care and Population Health, University College, London, NW3 2PF UK; Health Economics Research Group, Brunel University, London, UB8 3PH UK

**Keywords:** Primary Health Care, Exercise, Walking, Non-participation, Qualitative, Randomised controlled trial

## Abstract

**Background:**

Trials in primary care to increase physical activity (PA) typically experience poor recruitment rates and may not recruit those with lower PA levels and who are most in need of the intervention. Despite the well-publicised benefits of physical activity, the majority of adults in the UK remain inactive and, therefore, at greater risk of many health problems. Our aim was to investigate the reasons for non-participation in the PACE-UP trial, which is a primary care pedometer-based walking intervention. This is important for successful recruitment and retention in future PA trials and programmes.

**Method:**

We conducted semi-structured audio-recorded telephone interviews with 30 participants, aged 45–75 years, purposively sampled from those declining participation in the PACE-UP trial. Recruitment continued until data saturation and a demographically balanced sample was achieved. Interviews were transcribed verbatim, coded and subjected to thematic analysis.

**Results:**

Interviewees supported walking as suitable exercise for most people in this age group, recognised the importance of this type of research and general practice as an appropriate setting. Key reasons for declining were: the perception of being already ‘too active’; existing medical conditions; work; travel and other commitments. Less frequently cited reasons included reluctance to be randomised, the intervention’s duration, wearing a pedometer, perceived inappropriateness of trial literature and a preference for a different kind of PA or for a group activity.

**Conclusions:**

Whilst most interviewees perceived themselves to be sufficiently active, an important minority did not participate due to existing medical conditions and other commitments. Recruitment to future PA trials might be improved by tailoring activity to compensate for medical problems, and adapting PA interventions to fit around work and travel commitments. Ensuring that patient-targeted literature is succinct and inclusive and that equipment is user-friendly are also important. Primary care is seen as an appropriate setting for PA trials and programmes.

**Trial registration:**

ISRCTN98538934.

## Background

Despite the conclusive and well-publicised evidence of the benefits of physical activity (PA), PA trials typically have low recruitment, of between 6 [1] and 35 % [2], and investigators often struggle to recruit representative participants [3]. Investigating differences between participants and non-participants is important for determining trial generalisability and ensuring that the intervention targets appropriate populations. The external validity of a trial is hampered if those recruited do not represent those who would be targeted in a ‘real life’ setting [4]. Some reports suggest that participants are more active [5–7] and have better health [6, 8] than non-participants, but others have found that they have poorer health [5, 9]. The underlying *reasons* for non-participation have not been fully explored with quantitative data and are important for those designing community PA trials and evidence-based PA programmes.

The importance of ensuring that those most in need of PA interventions are effectively targeted cannot be over-stated. Adequate PA levels reduce the risk of many health conditions [10], whilst physical inactivity results in over 3 million preventable deaths per year worldwide [11]. Current UK PA guidelines for adults and older adults recommend at least 150 minutes of moderately intensive PA weekly, or 75 minutes of vigorous PA weekly, both in at least 10-minute bouts [10]. Recent surveys based on objective PA assessment suggest that fewer than 10 % actually achieve recommended levels (much lower than those self-reporting achieving them) [12]. Increasing PA is a key priority for Public Health England [13] and targets for delivering short PA interventions have recently been introduced into the primary care National Health Service (NHS) health checks offered to 45–74 year-olds [14].

The PACE-UP PA trial is a three-arm randomised controlled trial (RCT) aiming to increase walking to achieve public health PA targets in 45–75 year-old primary care patients. It compares three groups: (1) pedometer plus practice nurse support, (2) pedometer alone (delivered by post), and (3) usual care. Both intervention groups received a 12-week walking programme and an individualised PA diary. Both the postal and nurse intervention employed behaviour change techniques aimed at increasing PA from an individual’s baseline level and building lasting habits.

Potential trial participants were identified from seven south-west London (UK) general practices (GPs), representing diverse socio-economic and ethnic groups. Medical records were screened and those with a contraindication to increasing PA were excluded and a random sample of eligible participants were invited to participate by post. Medical reasons for exclusion included: at least three falls in the previous year or at least one fall in the previous year requiring medical attention; terminal illness; dementia or significant cognitive impairment; registered blind; new-onset chest pain, myocardial infarction, coronary artery bypass graft or angioplasty within the last 3 months; medical or psychiatric condition which the GP considered excluded the patient (for example, acute systemic illness such as pneumonia, psychotic illness). The protocol is available elsewhere [15].

### Aim

To investigate reasons for non-participation in a primary care-based PA intervention.

## Methods

Those declining trial participation were asked to complete a non-participant questionnaire (NPQ) designed to capture the key reasons for non-participation. These were categorised as: (1) I do not have time, (2) I cannot increase my PA, (3) I am not interested in increasing my PA, (4) I am already very physically active, (5) I am not interested in research, and (6) I do not want to be put in a group by chance. Space was provided for those completing the NPQ to provide reasons for non-participation not covered by these categories. Non-participants were asked if they could be contacted to discuss their reasons in more detail.

A purposive sample of those willing to be contacted was selected to provide men and women of varying ages, ethnicities and employment statuses from the initial six participating practices. They were contacted promptly on receipt of the NPQ to aid their accurate recollection of the trial literature and reasons for declining. To maximise participation we used focussed telephone interviews and gained permission for interviews to be audio-recorded. The topic guide (Appendix 1) was developed from the literature, qualitative findings from a preceding PA trial [16] and discussion between authors. Approximately 30 interviews were planned, with recruitment continuing until no new themes were identified and a demographically balanced sample had been achieved.

After obtaining informed consent for the interview, we asked open questions about what influenced their decision not to participate and their opinions of the trial information received. Responses given on their completed questionnaires were used as a starting point to further explore reasons for non-participation. They were asked broad questions about their perception of the trial design and invited to make any concluding comments.

### Data analysis

Audio-recordings were transcribed verbatim and checked for accuracy. After 10 interviews, researchers (RH, CV, TH) read the transcripts and discussed the interviews. Following the meeting, the interview technique was modified slightly to ensure that interviewees understood the trial randomisation process as several participants had appeared not to understand the question about whether being put in a group by chance had influenced their decision not to participate.

On completion of interviewing, transcripts were read and re-read for familiarisation by two researchers (RN, TH) who assigned codes, before a thematic framework was produced [17]. Coding discrepancies between researchers were resolved by discussion. The framework produced was informed both by a-priori issues, mostly related to trial design, and by emerging themes. Themes were refined further by discussion between authors and broader categories, encompassing several sub-themes, were generated. Authors also attempted to identify a main reason for non-participation for each interviewee based on the most frequently mentioned reason or the reason appearing to carry most weight. This was also agreed by consensus. Reasons for declining given by all NPQ respondents were also compared with those given at interview, to put our findings in a wider context and assess generalisability to all those declining.

### Ethics

This PACE-UP trial has been reviewed and given a favourable opinion by the London Research Ethics Committee (Hampstead) (12/LO/0219).

## Results

In total, 11,015 patients aged 45–75 years from seven south-west London GPs were invited to take part in the main trial. One thousand eight hundred and sixty (17 %) returned a form expressing interest in participation with 1023 eventually being randomised. We received no response from 6399 (58 %) and 2756 (25 %) returned a form declining participation, of whom 1140 (41 %) completed an NPQ. The total proportion of those who did not respond or declined to participate who completed an NPQ was 1140/9155 (12 %).

### Interview participants’ characteristics (Table 1 and Appendix 2)

**Table 1:** Interview participant demographic and main reason for non-participation summary table

Characteristic		Participant
		*n*
Age	45-95 years	15
	60-75 years	15
Sex	Male	14
	Female	16
Ethnicity	White	23
	Asian/Asian British	2
	Mixed/multiple ethnic groups	2
	Black/African/Caribbean/Black British	3
Employment	Retired	9
	Part-time	6
	Full-time	8
	Looking after home or family	1
	Student	1
	Other	5
Home owner status	Owner	25
	Rent from council or housing association	4
	Rent privately	1
Age of leaving education	19 years or over	15
	18 years	2
	17 years	2
	16 years or less	10
Main reason for declining from interview	Too active	18
	Medical problems	4
	Travel	3
	Work commitments	2
	Other commitments	1
	Equipment problems	1
	Does not want to be randomised	1

Fifty-five trial non-participants were telephoned between March and July 2013, 21 could not be contacted and 4 declined to be interviewed. Thirty trial non-participants representing the six initial participating practices were interviewed. Data saturation was achieved prior to completing 30 interviews, but we continued to 30 to ensure a more ethnically diverse sample and to achieve demographic balance.

### Thematic analysis of interview responses

#### Main reason for declining (Table 1)

For most interviewees there appeared to be a main reason for declining participation, which emerged from the transcript of the interview. This was consistent across gender, ethnicity and age groups. The majority (*n* = 18) said they were too active either because they felt their activity exceeded the trial’s target levels, or because these levels of activity meant that others would benefit more than they would from participating:

*‘*I'm a very active person. I dance, I do exercises, I run, I walk, I go to the gym. I've been pretty much active the whole of my life … so I don’t think I need it really’. (identification number (IDN)11)

‘What I understood is you are more interested in people who do less exercise or none at all and I do exercise almost every day’. (IDN27)

‘You would have been better putting your effort into somebody who was not so motivated’. (IDN22)

Less frequently cited main reasons included existing medical problems (*n* = 4), travel from home (*n* = 3), work/other commitments (*n* = 3), concerns about potential equipment problems (*n* = 1) and reluctance to be randomised (*n* = 1).

#### Further exploration of reasons for non-participation (Fig. 1 and Table 2)

**Fig. 1:**
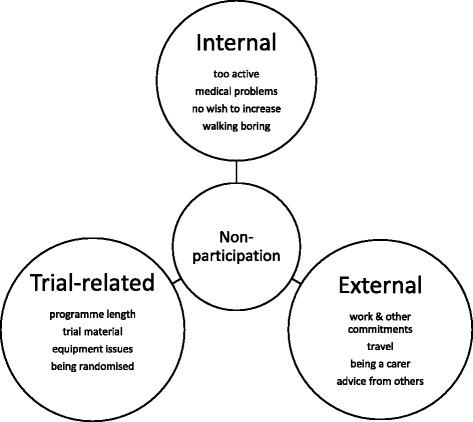
Illustration of the main reasons for non-participation which emerged from the interview data

**Table 2:** Summary of categories and themes – reasons for declining

Category	Sub-category	Theme	Quotes
Personal	Already active	Personal activity	‘I tend to walk quite a lot anyway, so I didn’t think a pedometer would probably be likely to increase my walking at all really’. (IDN12)
		Work activity	‘I actually work as a postman, so I do a hell of a lot of walking … and that was basically the reason that I didn't think I’d need to actually join the programme’. (IDN06)
	Medical problems	Stroke	‘I had the stroke in ’94. So that limited my walking’. (IDN07)
		Pain	‘If I walk for more than half an hour at a time, I get incredibly stiff and painful’. (IDN16)
		Heart condition	‘I’m always at the hospital seeing a cardiologist’. (IDN18)
		Multiple medical problems	‘I don’t need anything else going on to do with health … I certainly would have thought … that they would have thought, “oh, she wouldn’t want to do this because she’s got lots of other problems”’. (IDN18)
	No wish to increase activity	Not interested/does not like physical activity	‘And I don’t really like running … and I certainly won’t join a gym. I hate exercising’. (IDN02)
		Already doing enough	‘No, I think I do enough. I’m fine with what I do’. (IDN17)
	Not interested in walking	‘More interesting than walking’	‘Cycling’s nice, swimming … any form of recreation thing, like ice skating or horse riding or bicycle riding, anything like that … Walking’s quite boring’. (IDN02)
		Team sport	‘Well, it would be hard for you to organise team sports I should think wouldn’t it? I mean I used to play badminton quite a lot which I enjoyed’. (IDN19)
		Running	‘If anybody’s doing research into people that have had heart attacks and then trying to get back into running, that I’d be extremely interested in’. (IDN24)
	Not the right person	For younger people	‘You get to a stage in your life and you think, that’s it… I’m relaxing now. I exercise my mind instead’. (IDN02)
		For lonely people	‘These sort of things people take them up if they’re lonely and I’m not lonely’. (IDN18)
		For overweight people	‘Unless you were a really fat person, which I’m not’. (IDN18)
	Altruism		‘… an opportunity for someone else, you know, that it may be more useful to’. (IDN13)
External	Work commitments		‘It’s bad enough trying to get … a day off for a normal appointment*.*’ (IDN02)
			‘I just didn’t think I’d have time … because I know how important walking is, and I love walking, and if I have an hour or two free, I would prefer to walk than talk to the nurse’. (IDN21)
		Travel difficulties	‘If I had time, I’d love to be part of your research and go to the surgery and all the rest of it, but I think, actually … the awkwardness of the journey…’ (IDN22)
	Other commitments	Travel from home	‘I’m going away so much, I couldn’t really tie myself down to anything like that’. (IDN01)
		Caring for family member	‘I’m a carer for my father. I think most of it is just being there’. (IDN04)
		Chores/‘life’	‘I’ve got grandchildren. I’ve got a husband. I like to do my gardening. I’ve got a four bedroom house to keep clean. I feel my load is more than enough to keep me going’. (IDN08)
	Advice from others		‘I did mention it to my daughter actually and she said “that sounds crazy!” She said it’s not for me, so I didn’t go any further’. (IDN07)
Trial-related	Length of programme		‘It does sound a bit on the lengthy side doesn’t it really… some people could be put off by that’. (IDN10)
	Trial material	Too long	‘… there was a lot to read. Bullet points are good. Just make it simple’. (IDN19)
		Aimed at older people	‘I just remember thinking, actually, I don’t think I’m in that age group yet. It kind of seemed to be geared to people who really were in their 70s and over’. (IDN09)
	Equipment problems	Pedometer/accelerometer	‘Well, I mean I have actually used a pedometer but I wouldn’t sort of particularly want to do it for a week’. (IDN09)
	Randomisation	Did not like concept	‘I think if you’re doing research then you should be able to choose …within reason …what club you’re willing to join really’. (IDN13)
		Did not want to be in nurse support group	‘… I could probably commit to the other two groups, but possibly not to the nurse support’. (IDN09)
		Did not want to be in control group	‘Well … I couldn’t see the point of being in a group that did nothing’. (IDN04)
	Venue	Fitness-related better	‘If you’re going to do a fitness programme, you should do it in a fitness venue’. (IDN04)
		Does not like the GP surgery	‘I have to go there when I’m not well. I certainly am not going to go to the surgery when I’m well’. (IDN18)
	Walking environment	Boring	‘Walking’s quite boring. Unless you’re walking somewhere on an outing somewhere, you know, in the country or something, seaside. You should have more trips’. (IDN02)
		Wrong season	‘As the weather gets better, then I might go for a walk in the evening … it was really due to the seasons as well’. (IDN28)
	Preferred group		‘I think you get more encouragement if you are in a group’. (IDN05)
	Trial design		‘… that isn’t something I wanted to be part of I think I’d have found it incredibly boring’. (IDN18)

To further explore reasons for declining we categorised the themes that emerged from the interview data. We defined ‘internal’ reasons as those related to the participant themselves (for example, medical problems or personal preference), which may have precluded participation in any PA intervention, whether in a trial setting or not. ‘External’ themes related to the wider life of the participant (for example, work and family commitments) which again were unrelated to the specific nature of the intervention they were being offered. The ‘trial-related’ category identified reasons mentioned by participants that were explicitly related to the design of this intervention (for example, the use of a pedometer or the location of appointments). For each individual participant their reasons for non-engagement may be overlapping and relate to more than one of these categories; for example, one participant reported that regular work-related travel (an ‘external’ reason, which may have been problematic for increasing PA generally) prevented him from committing to an intervention that may require three appointments at his local surgery (a specific ‘trial-related’ reason).

##### Internal

This category identified all ‘internal’ reasons for non-participation, including being already active; medical problems (pain, heart conditions, stroke and multi-morbidity); no wish to increase activity; no interest in walking; feeling incorrectly ‘targeted’; and altruistic reasons. The dominant reason in this category was a belief in being already sufficiently active. When explored in more depth it seemed that on self-report many were achieving, with some significantly exceeding, the recommendations:

*‘*I basically run 10 ks one day and 15 ks the next, and I do that Monday, Tuesday, Wednesday. Thursdays I cycle. And then I run again Friday and Saturday. And then in summer I used to do 15 ks one day and 20 the next’. (IDN24)

‘… 5 days a week I swim, and I swim 1000 metres every day, I mean from Monday to Friday, and I walk every day’. (IDN27)

Of those citing medical reasons, it was less clear whether these problems constituted a definite contraindication, especially as those with pre-defined medical conditions contraindicating an increase in walking should have been excluded:

‘If I walk for more than 40 minutes at a time, or half an hour at a time, I get incredibly stiff and painful’. (IDN16)

A small number of people suggested that they did not enjoy PA, were not interested in walking or suggested a different activity or a team sport:

‘Can’t you take up archery or something?! If you had one of them I’d do that. You’ll have to get more interesting things (than walking)’. (IDN02)

##### External

This theme relates to factors ‘external’ to the potential participant, including work and other commitments; travel problems; being a carer and advice from others. Work and work-related travel were frequently given as reasons for not participating and many feared they could not make the necessary commitment:

‘The reason I said “no” to doing it, in the first place, was I travel a fair bit with my job, overseas, and I thought that might sort of hinder me doing the experiment sort of properly you know because obviously I’m here for a couple of weeks and I go off for 3 weeks’. (IDN15)

For one interviewee who worked a long distance away, travelling to appointments was an important reason for declining. Family and home life commitments, including caring roles, were also important reasons for feeling unable to participate:

‘I feel my load is more than enough to keep me going’. (IDN08)

We were interested in finding out whether advice from friends or family affected the decision not to participate as evidence suggests that presence or absence of social support influences PA levels [18]. Very few interviewees discussed participation and for those who did, it did not influence their decision, except for one interviewee whose daughter strongly advised that it was not appropriate to be increasing PA due to medical problems and this finalised the decision:

‘I did mention it to my daughter actually and she said “that sounds crazy!” She said it’s not for me, so I didn’t go any further’. (IDN07)

Reasons related to trial design included programme length; trial material; equipment problems; being randomised; the venue; the walking environment; the nurse interaction and the overall trial design.

For some interviewees the trial duration, at 3 months, was too long and it was difficult to commit for this period, although no-one cited this as a main reason for declining:

*‘*Yes, yes, because I’m travelling a lot, so 3 months is quite a long period for me. I may not be able to meet the nurse or the researcher at certain times if I’m out of town’. (IDN27)

A few felt that the trial literature was too long or appeared to be aimed at an older age group but for only one interviewee was this an important factor in the decision not to participate:

‘Well, you’re talking to a generation of oldies now who don’t accept that we’re old. We don’t feel old … and we don’t accept it, and I haven’t grown old like my parents did’. (IDN26)

One interviewee reported a previous negative experience with pedometers as the main reason for declining:

‘Well, I mean I have actually used a pedometer but I wouldn’t sort of particularly want to do it for a week’. (IDN09)

Several felt that not being able to choose their allocated group was a disadvantage. Some expressed reluctance to be in the control group:

‘I’m not sure, but I probably would say I wouldn’t want to be put in a group by chance. I would want to see that there was some positive outcome to whatever I was doing’. (IDN21)

Other trial design aspects noted by interviewees included: not having time to be in the nurse-support group; preference for a ‘fitness venue’ and reluctance to go to the GP surgery when well. Two interviewees expressed concern about walking as an exercise because the local walking environment was ‘boring’ and another that it was ‘the wrong season’ for walking outdoors.

Some expressed interest in a group intervention rather than one-to-one with a nurse, feeling that this would improve motivation and sociability:

‘The way to get people exercising is to get them together. Because motivation’s always a problem isn’t it? If you … oh, I can’t be bothered, I won’t do it today, but if someone else is going along …’ (IDN04)

#### Positive comments about the trial (Table 3)

**Table 3:** Summary of categories and themes – positives

Category	Sub-category	Theme	Quote
Trial design	Venue	Pleasant	‘Our doctor’s practice is lovely’. (IDN08)
		Convenient	‘If the study is conducted at the GP practice it would be very convenient for me because I live very nearby’. (IDN27)
		Appropriate	‘It seemed appropriate actually’. (IDN16)
	Structure	One to one is better	‘No, no, no, no. No, no, no. No. I get all tongue-tied in groups. And I certainly wouldn’t tell them anything personal or private’. (IDN02)
	Trial literature	Clear	‘It was all fine … clear and precise, so no issues with that at all really’. (IDN13)
Research	Important/good		‘It’s a good thing I suppose. I mean my husband’s had a triple bypass. And I’ve had cancer so you know … research is a good thing’. (IDN08)
	Interesting		‘I am interested in research. I just don’t think that I would have anything to contribute to this particular project, that’s all’. (IDN29)
Exercise	Positive about an exercise programme	Important/beneficial	‘I think it was a good thing to do, and it certainly made [name of partner who did participate] much more conscious of walking more, which is a good thing. Been beneficial for him I think’. (IDN16)
		Good for older adults	‘Well, I think it is a very good idea, especially for senior citizens, like myself, to be encouraged to take part in physical exercise, and for you to be interested in people’s welfare’. (IND01)
	Walking is appropriate		‘I think walking’s probably the best, you know, it’s the one most people can participate in’. (IDN21)

Many interviewees expressed a positive attitude towards physical activity and research and regretted not being able to participate.

Most interviewees approved of the choice of their GP surgery as the location for a PA intervention, describing their surgery as ‘lovely’, ‘pleasant’, ‘convenient’ and ‘appropriate’:

‘I think the GP practice is fine. It’s a nice sort of facility there’. (IDN14)

Most also did not object to meeting a nurse one-to-one and for some this was preferred to a group. In addition, many interviewees felt that the trial literature was clear and of an appropriate length:

‘Well, it was enough information, not too much, I think if you give people too much information they don’t read it do they?’ (IDN04)

Many interviewees stated that despite declining, they valued research, describing it as ‘important’, ‘beneficial’ and ‘interesting’:

‘If people don’t do any research we won’t know anything about anything, will we?!’ (IDN13)

Many interviewees were positive about exercise and interventions to increase walking in adults and older adults. Walking was generally thought to be an appropriate and inclusive activity:

‘As we get a bit older, walking is probably the best exercise we can all do, and we do most of probably’. (IDN13)

### Comparison of interview and NPQ responses (Tables 1 and 4)

**Table 4:** Comparisons of reasons for non-participation between all questionnaire responders and interviewees

Reasons^a^	All non-participant questionnaire (NPQ) responses (*N* = 1140)	Interviewees’ NPQ responses (*N* = 30)
	*n* (%)	*n* (%)
Did not answer ‘yes’ to any question	141 (12)	2 (7)
Already physically active	668 (59)	20 (67)
Do not have time	468 (41)	13 (43)
Cannot increase physical activity	225 (20)	6 (20)
Not interested to increase physical activity	208 (18)	5 (17)
Do not want to be randomised	113 (10)	3 (10)
Not interested in research	48 (4)	1 (3)

^a^Reasons for declining from NPQ (each non-participant could select one or more reasons from the list and for each question could answer: yes; no; not sure; or leave the question blank)

Although we found reasonable agreement between the answers given on the NPQ and the reasons for non-participation expressed at interview, some of our interviewees had not answered the question about why they decided not to participate and others ticked ‘yes’ to several options so it was only through discussion at interview that a main reason could be elucidated (see Table 1).

Table 4 shows the reasons given for non-participation on the NPQ for all those completed (1140) and for the 30 who were interviewed. The interview findings substantially reflect the responses of the whole group, with the majority declining due to being already very physically active or not having sufficient time. This suggests that our interview sample was reasonably representative of those completing the NPQs.

## Discussion

### Principal findings

The main reason for non-participation established at interview was a perception of being already too active for the trial. Other important reasons included medical problems, work and other commitments and travel from home. Despite declining, almost all interviewees were positive about the trial, aware of the benefits of PA, the importance of research and supported primary care as a venue for such programmes. The design of the trial and intervention was not stated as a key reason for declining to participate.

### Strengths and limitations

This study represents an innovative attempt to systemically explore the reasons for non-participation with a purposive sample of those who were potentially eligible but declined. Currently, there is limited work in this area and the findings are of interest to those planning PA trials and may be of interest to policy-makers. Our aim was to further understand the reasons for declining participation to enhance recruitment to future trials and exercise programmes. We were also able to explore non-participants’ perception of the trial design and research in general. This sample spanned six out of seven of the practices involved and included both genders, a range of ages, ethnicities, employment and educational backgrounds. The telephone interviews allowed in-depth exploration of the reasons for non-participation that was not possible from a questionnaire alone and allowed us to compare the interview findings with the NPQ responses from non-participants. Their positive comments also broadly reflect those made by trial participants who have been interviewed in a separate study [19].

The main study limitation is that the findings are based on a self-selected group of those who both returned the NPQ and agreed to be interviewed about their reasons for non-engagement. It would have been valuable to compare the responses of those actively declining with those of a sample of the over 6000 people who did not respond at all, but we did not have ethical approval to contact this latter, unrepresented, group.

Our categorisation of reasons into ‘internal’, ‘external’ and ‘trial-related’ provides a useful, simple framework for exploring the results. However, we accept that these categories are not entirely distinct from one another and reasons for declining for an individual are nuanced and overlapping. Our analysis suggests that the major reasons for declining to participate in the study were linked to the personal and social environments of individuals rather than the characteristics of the trial itself. Of particular interest is the large proportion declining because they felt themselves to be ‘too active’ to participate. It is not clear if these individuals would have been excluded in any case on the basis of their pre-existing activity levels and, therefore, their decision to decline may have been entirely appropriate. Also, despite our attempt to sample interviewees from non-white British backgrounds, these groups are under-represented when compared to the ethnic diversity of the population.

The NPQ sample represents 1140/9913 (12 %) of all those who declined participation or did not respond. The substantive similarities in main reasons for declining between the whole NPQ sample (*n* = 1140) and the 30 who were interviewed is reassuring. Whilst our findings are unlikely to be generalisable to all not wanting to take part, they do provide novel insight into the reasons given by significant numbers for not participating in a PA trial. To what extent these findings can be applied to those declining participation in PA programmes outside a trial setting is not certain; while it seems logical that some of the reasons mentioned, for example, work, travel and family commitments, might also present barriers to increasing PA outside a trial setting, findings should be generalised with caution.

### Comparisons with existing literature

A body of work exists which explores barriers to PA generally but fewer studies have attempted to explore the reasons for non-participation in PA trials specifically. Some of the reasons may be consistent across both PA generally and PA trials and we certainly found that many of the reasons given by our interview participants are in line with those emerging from the wider literature about PA participation.

Declining participation due to being already sufficiently active is consistent with the existing PA trial-related literature [20, 21] and also the literature about PA more generally [18, 22, 23]. It is important to note that objective measurement of PA reveals that most people over-estimate their activity levels [12] and that their assessment of their personal activity levels is likely to be influenced by a social context [18, 24]. However, this interview series allowed activity levels to be explored in more detail and revealed that, at least on self-report, this was a relatively active cohort for some of whom the trial may not have been appropriate.

Declining participation in PA programmes or trials due to medical problems, including pain, is also consistent with previous work [9, 18, 20, 23, 25], particularly in studies involving older participants [21, 26]. Lack of time due to work and other commitments has also been identified as an important reason for non-participation in PA trials [20, 21, 27] as well as PA more generally [18, 24, 28–30], particularly in younger and middle-aged people [26]. Other reports suggest that social support can influence PA levels outside a trial setting [18, 24]; however, this did not emerge prominently from this trial-related interview series. A lack of interest in PA has also been reported in the literature as a reason for declining participation in both PA generally [18, 22–24, 30] and in PA trials [20] but travel away from home has not been reported prominently. This may reflect the high proportion of our interviewees still in full- or part-time work and the diverse population of south-west London, some of whom spend extended periods abroad.

### Implications for research and practice

Our findings have important implications for those planning PA trials and may be useful in other contexts, for example in the commissioning of community PA programmes. As the cohort we interviewed appeared relatively physically active, it may be necessary to tailor some interventions to maintaining, rather than increasing, activity. This is particularly important to prevent the well-recognised decline in PA that occurs with ageing [12]. Equally, education about the levels of activity that optimise health gain may prevent potential participants from declining due to over-estimation of their actual levels of activity. Measurements using pedometers or accelerometers provide a possible alternative approach to validating PA levels and although we recognise that they miss some activities such as swimming and underestimate others, such as cycling, they do measure walking accurately, which is the predominant PA type in this age group and was the focus of this trial.

Lack of time was an important reason for non-participation in this trial and is a common finding in studies exploring non-participation in PA more generally [18, 22–24, 28–30], so it may be helpful to reiterate that activity can be broken up into 10-minute bouts throughout the day (this can also help those limited by pain or disability). Tailoring interventions for an individual’s travel and work commitments and for their specific health problems may also increase uptake. Promotional material should explicitly state that pre-existing medical conditions do not necessarily prevent participation and dispel myths about the risks of moderate-intensity exercise. Indeed, none of the medical reasons for non-participation given by interviewees were contraindications to moderate-intensity activity and may, in fact, be positive indications for increased PA. Information about the value of PA, particularly walking, for many different health conditions should be emphasised in the invitation to participate.

Specifically related to trial recruitment, it is important to ensure that patient-targeted literature is inclusive and equipment is as user-friendly as possible. RCTs inevitably involve randomisation, but emphasising that in some trials (including PACE-UP) the control group can receive the intervention at the end of the trial may help recruitment. Those for whom travel difficulties were a reason for non-participation might respond well to an option for a remote consultation, for which there is increasing interest in primary care [31].

## Conclusions

A perception of being already sufficiently active was the main reason given by interviewees for non-participation in the PACE-UP trial. Other reasons included medical problems, work and other commitments and travel from home. An awareness of these reasons may be of use in other contexts, such as PA programme planning.

Despite declining, most interviewees were supportive of the aims of the trial and felt that primary care was an appropriate and convenient location for delivering a walking-based PA intervention. Their positive comments broadly reflect those made by trial participants who have been interviewed in a separate study.

## Consent

The ‘non-participants’ interviewed in this study were a sample of non-participants from a RCT. When they were approached for the trial they indicated in writing a willingness to be sent a questionnaire to complete, including reasons for non-participation, and on this questionnaire they indicated in writing that they were willing to be contacted by a researcher for a telephone interview to discuss in more detail their reasons for non-participation. They provided telephone contact details on the returned questionnaire, so that this could be arranged.

At the start of the interview the participants were informed that the interview would be audio-recorded if they consented to this. With their consent, the machine was then switched on and their verbal consent for the interview and audio-recording of this was repeated and captured on the audio-recording.

The methods for these non-participant interviews were approved by the Research Ethics Committee for the trial, as detailed in the manuscript. The same methods for consent were used in our previous PACE-Lift trial to capture reasons for non-participation from trial non-participants; this is published in a sister BMC journal: Rogers et al. *BMC Geriatrics* 2014; 14:46 [21].

## Appendix 1

### Telephone interview schedule for non-participants

Introduction

Clarify purpose of interview, gain verbal consent and confirm anonymity and confidentiality.

Opening questions

1. What did you think of the information that we originally sent you about the PACE-UP study?
2. Can you tell me a bit more about what influenced your decision not to take part in the study?
3. Did you discuss participating in research with anyone else?

Reasons for not participating

Using the completed questionnaire, explore the reasons already given, including:

1. I do not have time
2. I cannot/am not interested in increasing my physical activity
3. I am already very physically active
4. I am not interested in research
5. I do not want to be put in a group by chance

Additional possible reasons for not participating

Offer a number of other pre-defined reasons for non-participation and explore further any positive responses:

1. Lack of time
2. Unable or nor interested in increasing PA
3. Already active
4. Not interested in research
5. Do not want to be put in a group by chance
6. Length of programme
7. Travel difficulties
8. Wearing a physical activity monitor
9. Unpleasant/unsafe walking environment
10. Programme is not relevant to you
11. Programme is not for your age group
12. Programme would clash with work/being away from home
13. Medical problems prevent participation

Trial design questions

1. Venue
2. Exercise type
3. Group activity
4. Anything else that would have facilitated participation?

End

Summary and invite any final comments

## Appendix 2

**Table 5:** Anonymised interview participant demographics and main reason for trial non-participation

ID number	Employment status	Home ownership	Age left full-time education	Main reason for non-participation from interview	Reason(s) for non-participation from questionnaire
01	Retired	Owner	19 or over	Travel abroad (leisure)	Too active
02	Part-time	Rent from council or housing association	16	Work commitments	Not enough time, cannot increase activity, not interested in increasing activity, not interested in research
03	Other	Rent from council or housing association	15	Too active	Too active
04	Looking after home or family	Owner	19 or over	Too active	Not enough time, too active
05	Part-time	Owner	14 or under	Work commitments	No reason given
06	Full-time	Rent from council or housing association	16	Too active	Too active
07	Retired	Owner	14 or under	Medical problems	No reason given
08	Retired	Owner	16	Other commitments/ chores	Not enough time
09	Part-time	Owner	19 or over	Equipment problems	Not enough time, does not want to be randomised, does not want to wear pedometer
10	Full-time	Owner	16	Too active	Too active
11	Part-time	Owner	19 or over	Too active	Too active
12	Full-time	Owner	19 or over	Too active	Too active
13	Retired	Owner	15	Too active	Cannot increase physical activity, too active
14	Full-time	Owner	18	Too active	Medical problems, too active
15	Part-time	Owner	17	Travel abroad (work)	Not enough time
16	Other	Owner	19 or over	Medical problems	Cannot increase activity, medical problems
17	Student	Rent from council or housing association	16	Too active	Too active
18	Retired	Owner	16	Medical problems	Not enough time, cannot increase activity, too active, does not want to wear pedometer
19	Retired and Other	Owner	19 or over	Too active	Not enough time, too active
20	Full-time	Owner	17	Too active	Not enough time, too active
21	Part-time	Owner	19 or over	Too active	Not enough time, too active, work commitments
22	Full-time	Owner	19 or over	Too active	Not enough time, too active, work commitments
23	Retired	Owner	19 or over	Too active	Cannot increase activity, not interested in increasing activity, too active
24	Other	Owner	19 or over	Too active	Too active
25	Full-time	Owner	19 or over	Too active	Not enough time, not interested in increasing activity, too active, does not want to be randomised
26	Other	Owner	19 or over	Does not want to be randomised	Does not want to be randomised, does not consider themself ‘old’
27	Other	Owner	19 or over	Too active	Too active
28	Full-time	Rent privately	19 or over	Travel abroad (work)	Not enough time, not interested in increasing activity, too active
29	Retired	Owner	16	Too active	Not enough time, not interested in increasing activity, too active
30	Retired	Owner	18	Medical problems	Cannot increase activity, medical problems

## Abbreviations

GP: general practice/general practitioner
IDN: identification number
NPQ: non-participant questionnaire
PA: physical activity
PACE-UP: pedometer and consultation evaluation-UP
RCT: randomised controlled trial
UK: United Kingdom
